# Anomalous collapses of Nares Strait ice arches leads to enhanced export of Arctic sea ice

**DOI:** 10.1038/s41467-020-20314-w

**Published:** 2021-01-04

**Authors:** G. W. K. Moore, S. E. L. Howell, M. Brady, X. Xu, K. McNeil

**Affiliations:** 1 Department of Physics, University of Toronto, Toronto, Canada; 2 Department of Chemical and Physical Sciences, University of Toronto Mississauga, Mississauga, Canada; 3 Climate Research Division, Environment and Climate Change Canada, Toronto, Canada

**Keywords:** Climate-change impacts, Cryospheric science

## Abstract

The ice arches that usually develop at the northern and southern ends of Nares Strait play an important role in modulating the export of Arctic Ocean multi-year sea ice. The Arctic Ocean is evolving towards an ice pack that is younger, thinner, and more mobile and the fate of its multi-year ice is becoming of increasing interest. Here, we use sea ice motion retrievals from Sentinel-1 imagery to report on the recent behavior of these ice arches and the associated ice fluxes. We show that the duration of arch formation has decreased over the past 20 years, while the ice area and volume fluxes along Nares Strait have both increased. These results suggest that a transition is underway towards a state where the formation of these arches will become atypical with a concomitant increase in the export of multi-year ice accelerating the transition towards a younger and thinner Arctic ice pack.

## Abstract (web summary)

Ice arches that form along Nares Strait, which separates Greenland and Ellesmere Island, act to reduce the export of thick multi-year ice out of the Arctic. Here, we show that there has been a recent trend towards shorter duration arch formation that has resulted in enhanced transport of ice along the strait.

## Introduction

**Fig. 1:**
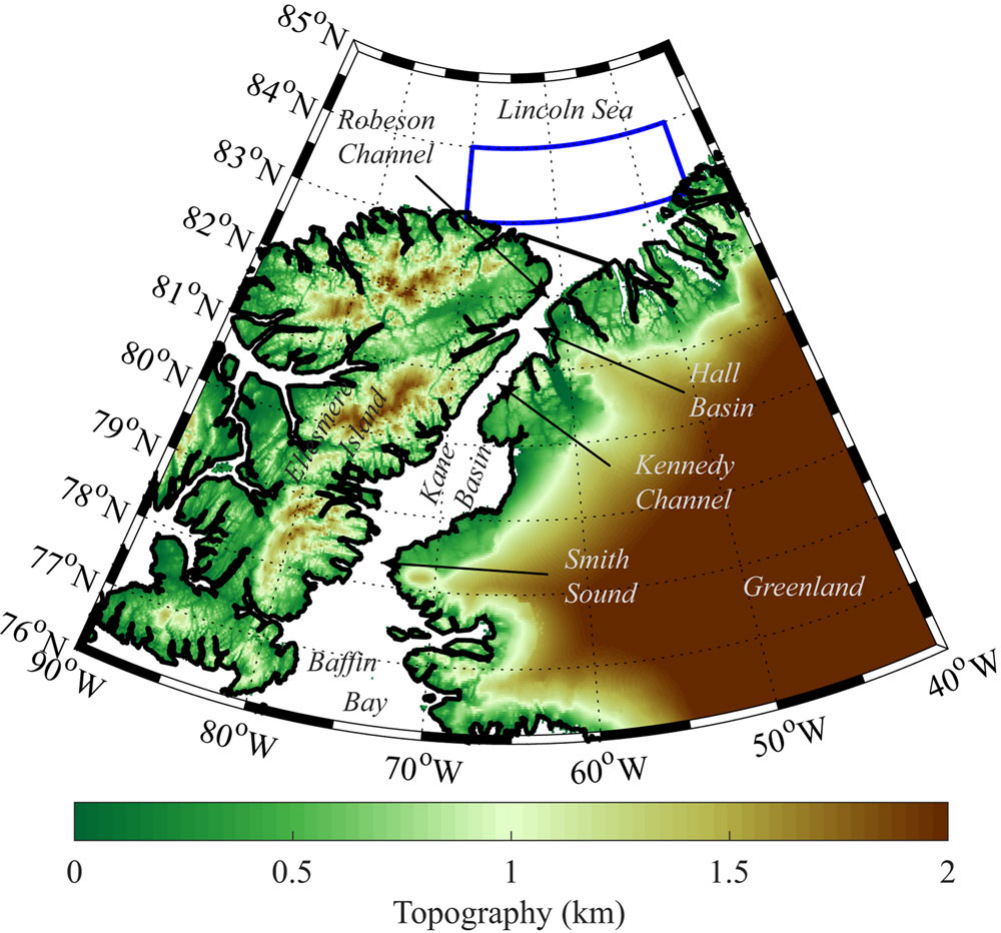
Topography (km) and place names in the Nares Strait region. The location of the fluxgate used to calculate the ice area and ice volume fluxes is indicated by the thick black line. The domain used to characterize the sea ice thickness of the Lincoln Sea is indicated by the blue polygon.

Along Nares Strait, the channel that separates north Greenland from Ellesmere Island, please refer to Fig. 1 for place names in the region of interest, ice arches typically form each winter at both its northern and southern ends^1,2^. The formation of either of these arches results in the cessation of ice transport from the Lincoln Sea southwards towards Baffin Bay and the subpolar North Atlantic^1,3^. The oldest and thickest sea ice in the Arctic is found to the north of Nares Strait^4–6^ and as a result, the formation of these arches, as well as ones that form along channels through the nearby Canadian Arctic Archipelago^7^ (CAA), contribute to the cessation of the transport of this important ice-class out of the Arctic^1,8^. For the period 1997–2009, the southern arch formed most winters while the northern arch formed during ~50% of the winters^2,9^. During the winter of 2007, neither arch formed resulting in annual ice area and volume fluxes that were twice as large as the corresponding climatological means over 1997–2009^2^.

The cessation of ice transport down Nares Strait contributes to the formation of the Arctic’s largest and most productive polynya, the North Water, at its southern end in the vicinity of Smith Sound^10,11^. In addition, climate models suggest that the area to the north of the Lincoln Sea will be the last to lose its perennial ice cover^12,13^ thus providing an important refuge, referred to as the Last Ice Area, for ice-dependent species^14,15^. The stability of these arches is a function of the thickness of the ice^16^ and there is a concern that the thinning of the Arctic ice pack may negatively impact their stability resulting in an acceleration in the loss of multi-year ice from the Arctic as well as impacting the ecosystems of the North Water Polynya and the Last Ice Area^2,14^.

We show that in addition to the previously identified early collapse of the northern ice arch in May 2017^17^, this arch failed to develop during the winters of 2018 and 2019. In contrast, we report that the southern ice arch was only present for a short period of time during the winter of 2018. The winter of 2019, like the previously documented winter of 2007^2^, was one in which no ice arches formed along Nares Strait. We furthermore show that there has been a recent increase in both the ice area and ice volume flux along Nares Strait as compared to the period from 1997 to 2009^2^. Over the period for which we have observations, 1997–2019, there has been a statistically significant trend towards shorter duration of ice arch formation each winter.

## Results

### The May 2017 Collapse

**Fig. 2:**
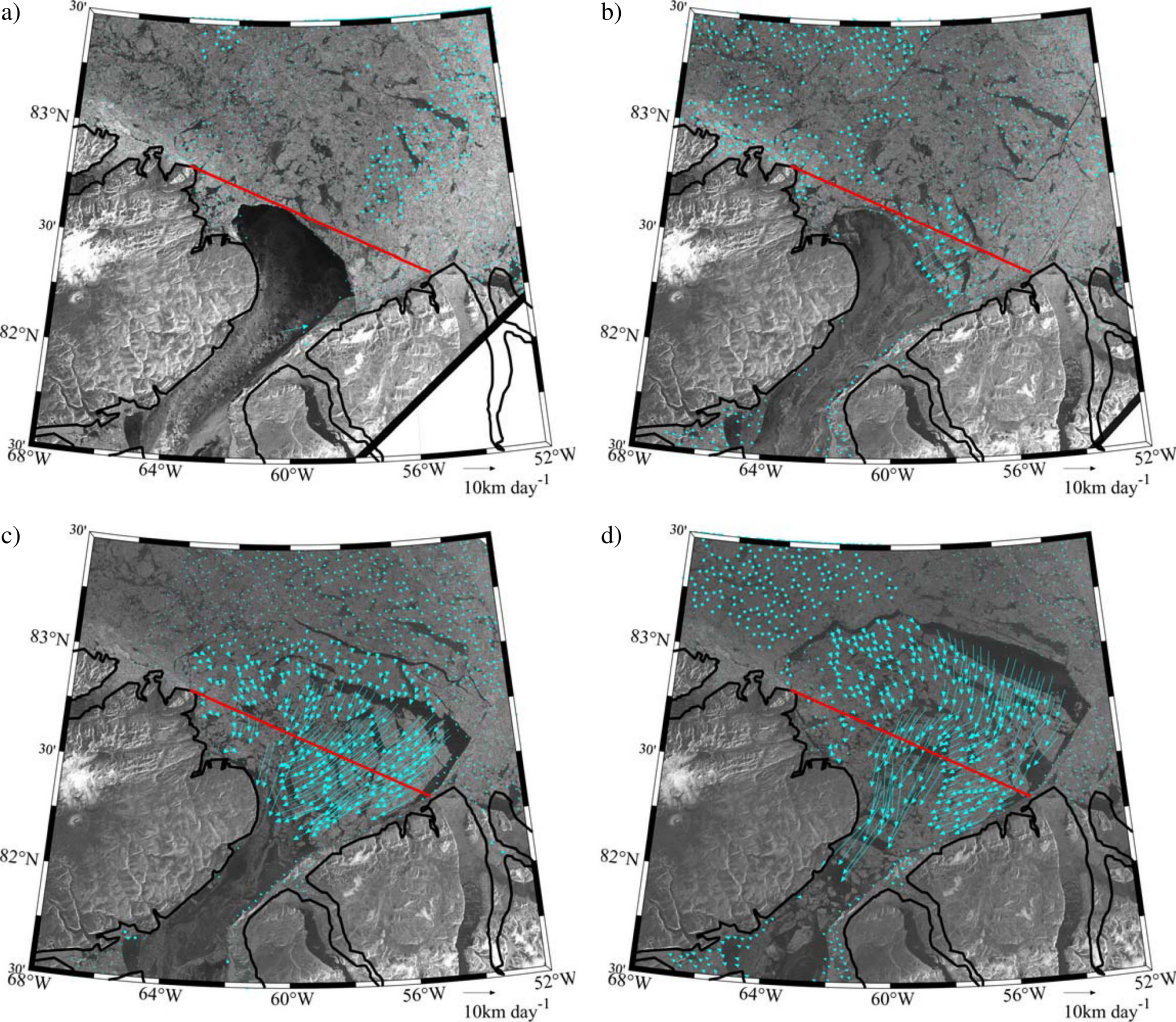
Collapse of the Lincoln Sea Ice Arch during May 2017. Sentinel-1 SAR satellite images and derived sea ice motion vectors (km day^-1^) on: **a** May 8, 2017 at 12:55 GMT; **b** May 10, 2017 at 12:40 GMT; **c** May 12, 2017 at 12:32 GMT; and **d** May 14, 2017 at 12:08 GMT. The southern Lincoln Sea flux gate used to calculate the ice area flux is shown in red.

During the winter of 2017, the northern ice arch collapsed in early May with thin ice in the Lincoln Sea hypothesized as contributing to the earliest collapse in the admittedly short record^2,17^. Fig. 2 shows the sea ice state of the northern Nares Strait and southern Lincoln Sea region during the period of the arch collapse from Sentinel-1 Synthetic Aperture Radar (SAR) imagery. Also shown are sea ice motion vectors, derived by feature tracking of sequential pairs of Sentinel-1 images using a technique described in the Methods Section^18^. On May 8 (Fig. 2a), the arch can be seen as the boundary between the thick multi-year ice to its north and the recently formed thin ice to its south. No significant ice motion was observed on this date, maximum ice velocities <1 km/day, indicating that the arch was stable. Two days later on May 10 (Fig. 2b), the arch had begun to collapse, as evidenced by southward movement of ice across the flux gate with maximum velocities ~5 km/day. Over the next 4 days (Fig. 2c, d), the arch fully collapsed resulting in ice velocities as large as 25 km/day that transported multi-year ice floes southwards into northern Nares Strait.

### Ice Area Flux

**Fig. 3:**
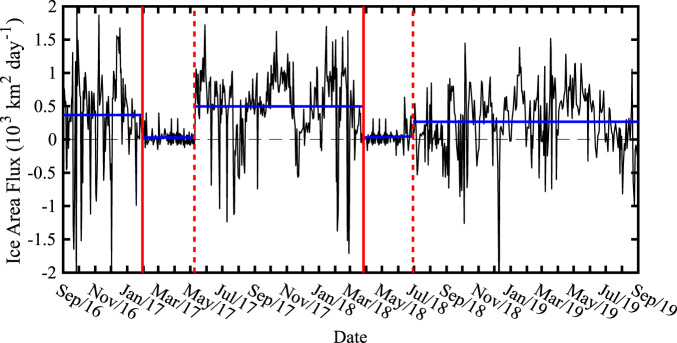
Daily ice area flux (10^3^ km^2^ day^-1^) across the southern Lincoln Sea flux gate. The vertical solid red lines represent the best available estimates for the onset of the stoppage of ice motion along Nares Strait during 2017 and 2018 with the dashed red lines representing the best available estimate of the end of the stoppage during 2017 and 2018. The average ice area flux over various periods of interest are indicated by the blue lines. All data based on Sentinel-1 satellite-derived sea ice motion vectors.

Using the Senintel-1 sea ice motion data across the flux gate indicated in Figs. 1 and 2, for the period of its availability, 2016–2019, it is possible to derive ice area flux data time series for Nares Strait that are similar to those reported for the 13-year period from 1997 to 2009^2^. Please refer to the Methods Section for more information. Fig. 3 shows this time series with the sign convention that southward/northward ice motion results in a positive/negative ice area flux. Although the flux is on average positive, it is highly variable in time with frequent brief instances where the flux is negative, i.e., northward ice motion. This high-frequency variability has been noted previously with acoustic Doppler current profiler data^11^ from the region and is consistent with the regional winds that show frequent reversals in direction^17,19^.

A noticeable reduction in the magnitude of the ice area flux occurred around January 30, 2017 (Fig. 3). Sentinel-1 imagery, not shown, indicated that the reduction of the ice area flux was associated with the formation of the northern ice arch. Low ice area fluxes persisted until the collapse of the ice arch around May 10 (Fig. 2). For the period from September 1 2016 to January 30, 2017, the average ice area flux was 366 km^2 ^day^−1^, while for period of the 2017 ice arch, it was 31 km^2^ day^−1^. After the collapse, the magnitude of the ice area flux was again large and highly variable until the end of March 2018 when a large reduction in magnitude also occurred. For the period of May 11 2017 to March 28, 2018, the average ice area flux was 496 km^2^ day^−1^. After March 28, 2018, the magnitude of the ice area flux remained small, an average of 46 km^2^ day^−1^, until late June 2018 when another transition to large magnitude and highly variable ice area flux occurred that persisted until the end of August 2019. For this period, the average ice area flux was 265 km^2^ day^−1^.

**Fig. 4:**
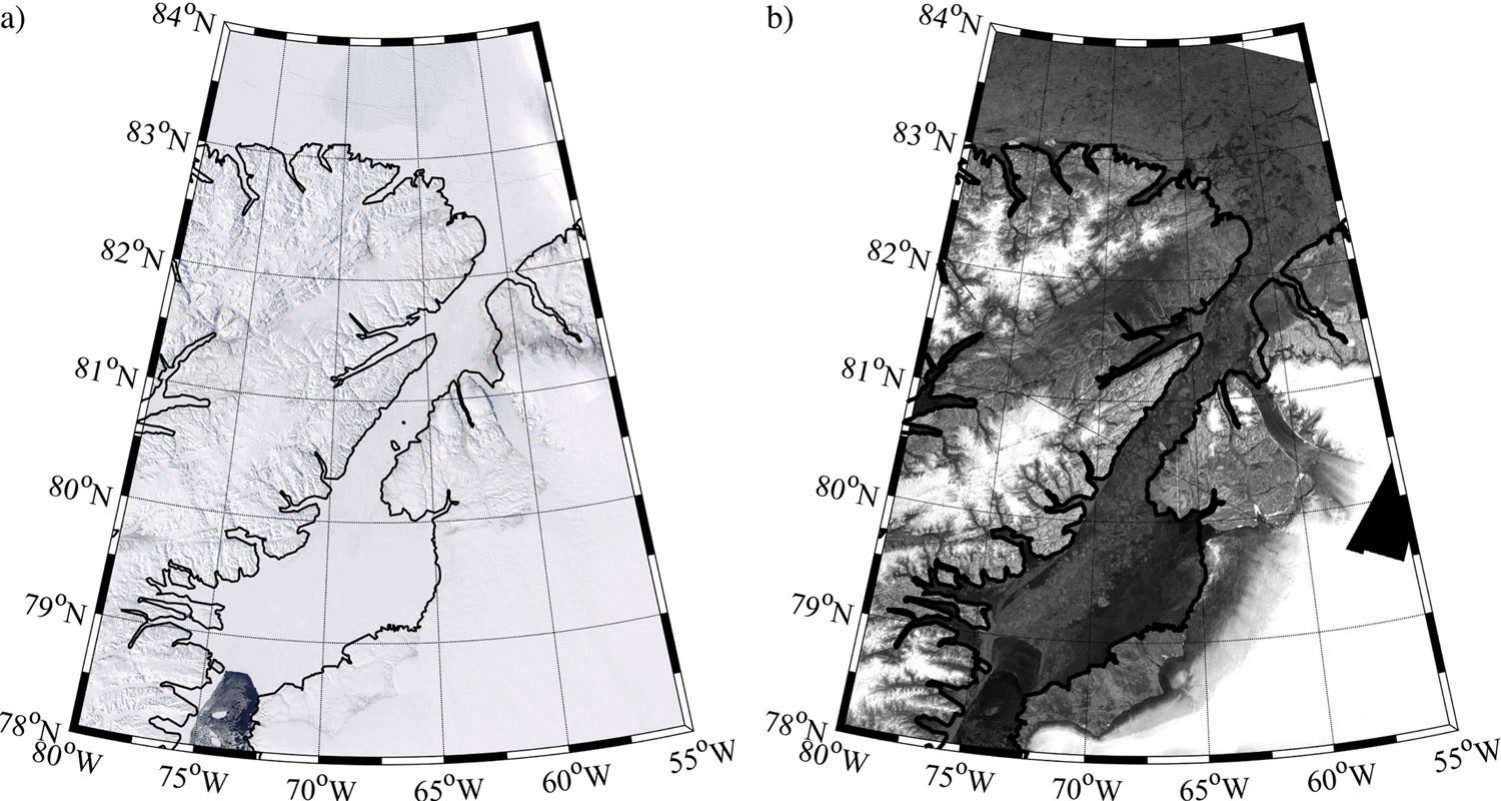
Nares Strait sea ice conditions during May 2018. **a** MODIS true-color satellite and **b** Sentinel-1 SAR satellite imagery from May 2, 2018.

Figure 2 and previous work^17^ indicate that the period of reduced ice area flux during 2017 was the result of the formation and subsequent collapse of the northern ice arch. MODIS true color and Sentinel-1 imagery during the 2018 period of low ice area flux, an example of which is shown in Fig. 4, show no evidence of a northern ice arch along with the presence of multi-year ice along Nares Strait with a southern ice arch along Smith Sound. In 2019, we interpret the absence of a period of reduced ice area flux (Fig. 3) as evidence that neither type of ice arch formed along Nares Strait during this winter.

**Fig. 5:**
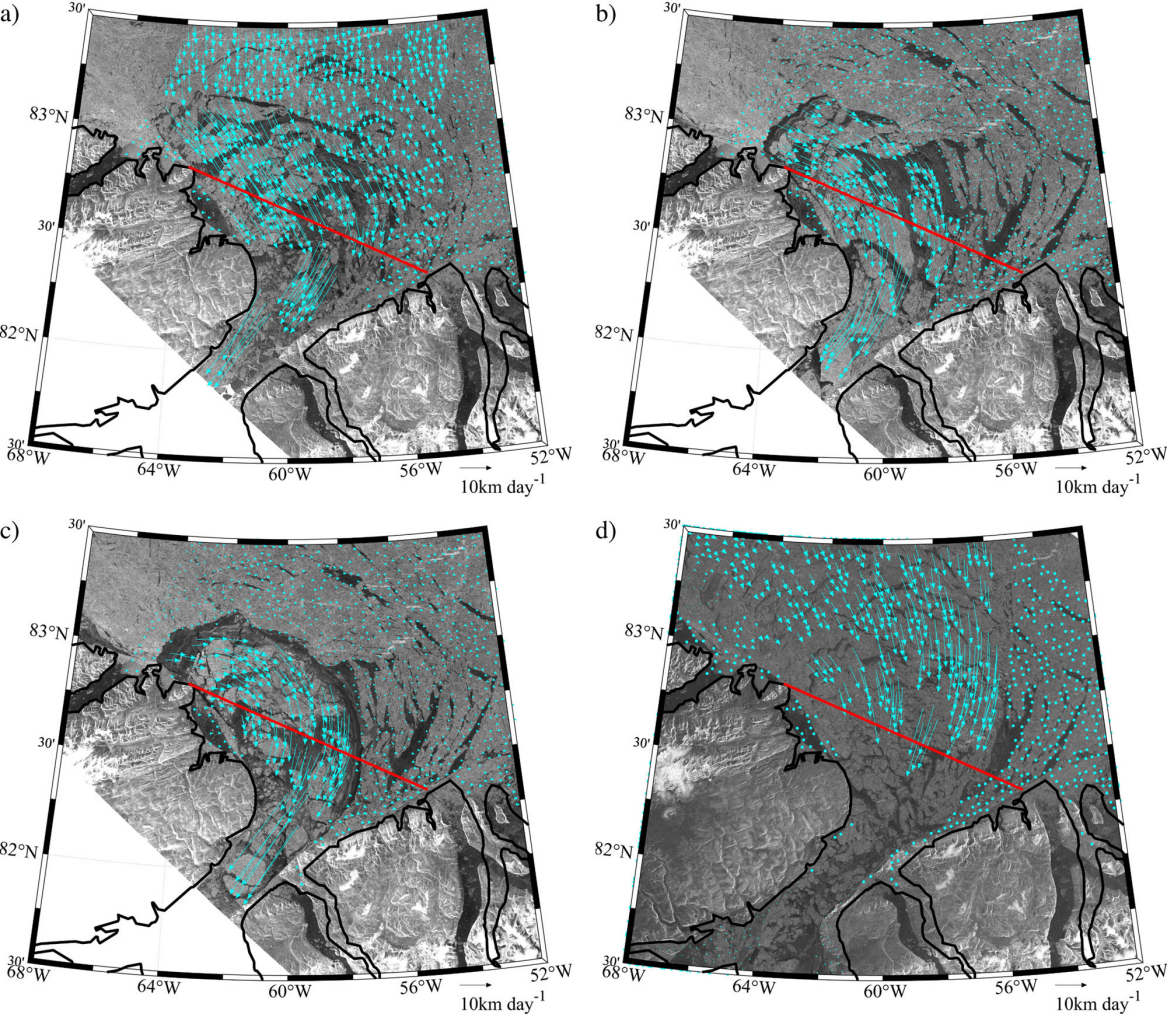
Lincoln Sea ice motion during 2019. Sentinel-1 SAR satellite images and derived sea ice motion vectors (km day^-1^) on: **a** January 14 2019 at 18:55 GMT GMT; **b** February 7 2019 at 18:55GMT; **c** February 19 2019 at 18:55 GMT; and **d** March 27 2019 at 12:33 GMT. The southern Lincoln Sea flux gate used to calculate the ice area flux is shown in red.

There were reports, based on visible satellite imagery, of the existence of the northern ice arch during the winter of 2019 and its subsequent collapse in March^20^. Indeed Sentinel-1 imagery (Fig. 5) does indicate the presence of an arch-like structure during the winter of 2019. However the associated ice-motion data indicates, in agreement with the ice area flux data (Fig. 3), that this structure was unstable and never resulted in the cessation of ice motion along Nares Strait.

**Fig. 6:**
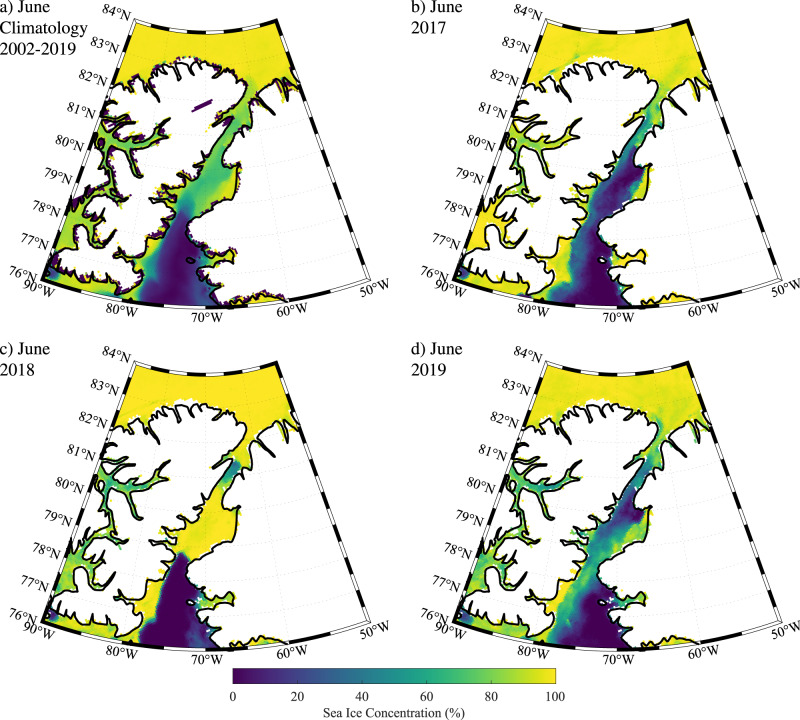
Sea ice concentration (%) along Nares Strait from AMSRE/2 satellite data during June. **a** climatology 2002–2019; **b** 2017; **c** 2018, and **d** 2019.

These conclusions are consistent with the monthly mean sea ice concentration based on passive microwave data from AMSR-E and AMSR2^21^. The climatology for June (Fig. 6a) indicates that the ice cover over the Lincoln Sea is typically close to 100%, while that along Nares Strait is lower at 60-80%. To the south of Nares Strait, over the North Water, ice cover is close to 0%. During June 2017 (Fig. 6b), after the collapse of the northern ice arch, ice cover along Nares Strait is lower than the climatology with open water along the eastern coast of the strait and higher ice concentrations to the west that is consistent with coastal downwelling and southward ice and ocean velocities associated the climatological northerly winds^22–24^. In contrast, during June 2018 (Fig. 6c) 100% ice cover was present along much of Nares Strait to the north of Smith Sound, a result consistent with a cessation of ice motion as a result of the presence of a southern ice arch. The situation during June 2019 (Fig. 6d) is similar to that during June 2017 and again is consistent with southward ice transport.

### Ice Volume Flux

**Fig. 7:**
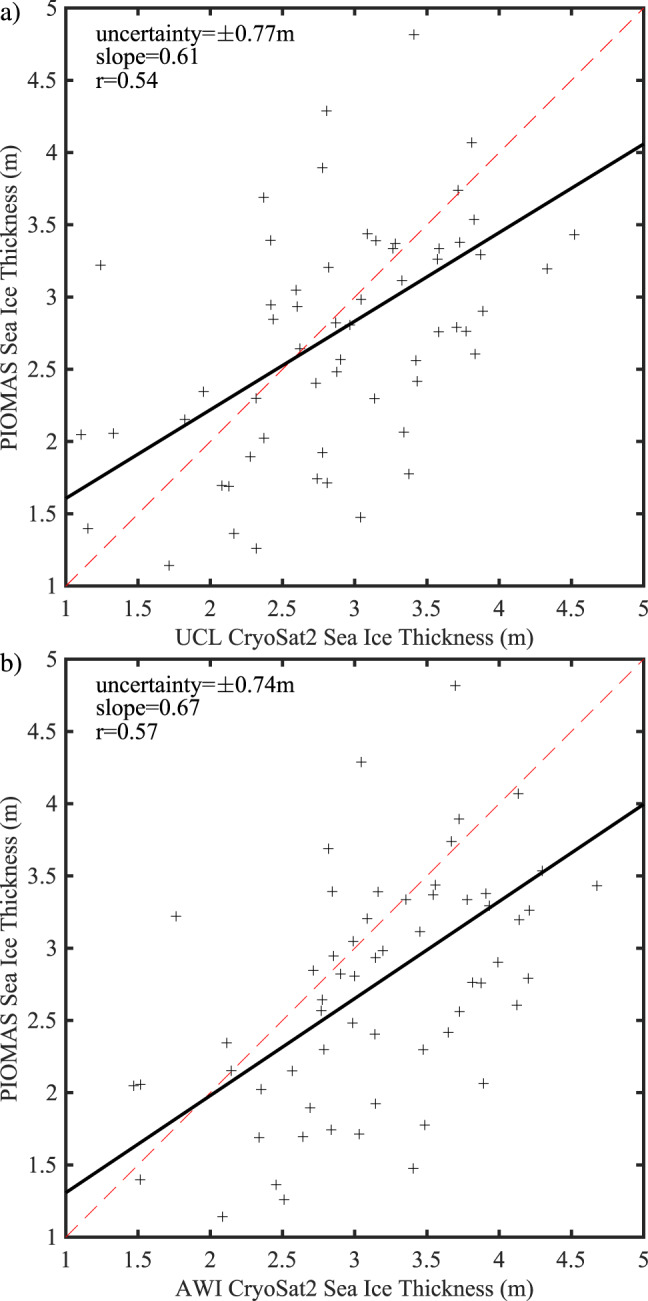
Observed and modeled sea ice thickness for the Lincoln Sea region 2010–2019. Scatterplots of the observed monthly mean: **a** University College London (UCL) and **b** Alfred Wegener Institute (AWI) CryoSat2 satellite data versus sea ice thickness from the PIOMAS model.

The combination of the ice area flux data for 2016–2019 presented herein with a previous record from 1997 to 2009^2^, allows one to examine the changes in the characteristics for the annual mean, defined over the period from September 1 to August 31 of the following year, sea ice transport along Nares Strait over the past 23 years. Annual mean ice thicknesses from the PIOMAS sea ice reanalysis^25,26^ are also used to derive ice volume fluxes. In the vicinity of Nares Strait, PIOMAS has a horizontal resolution of approximately 20 km^26^. PIOMAS has a recognized tendency to underestimate the thickness of thick ice and overestimate the thickness of thin ice^25,27,28^. Figure 7 compares the monthly mean sea ice thickness for two satellite-based retrievals, AWI CryoSat2^29^, and UCL CryoSat2^30^, against the monthly mean PIOMAS sea ice thickness data for a representative area of the Lincoln Sea shown in Fig. 1. The agreement between PIOMAS and the retrievals is consistent with previous work^12,25,28^.

**Fig. 8:**
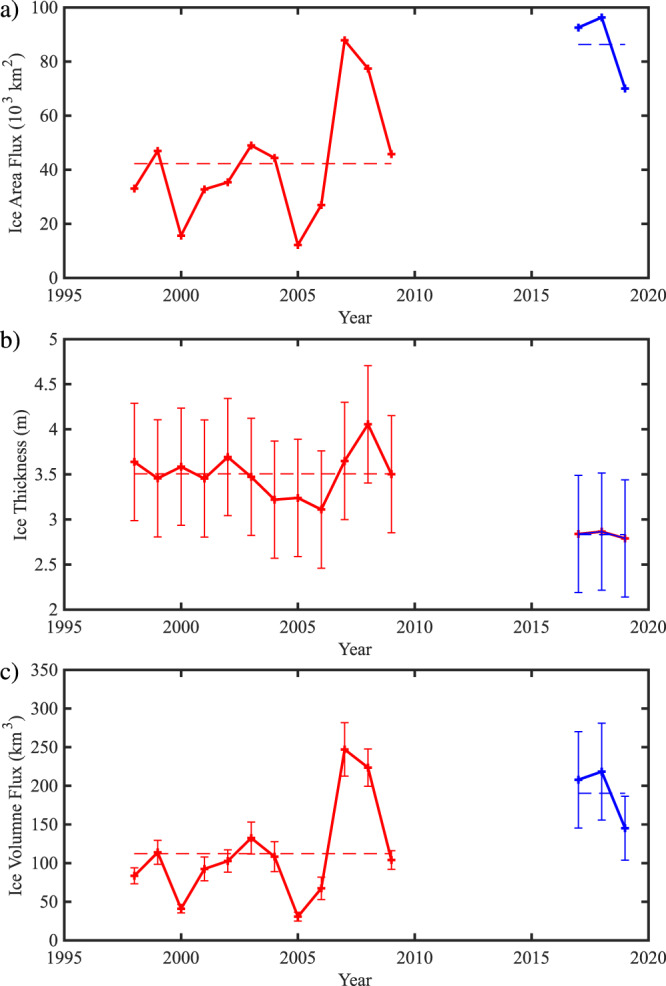
The changing nature of the ice flux through Nares Strait. **a** The annual mean ice area flux (103 km^2^) through the southern Lincoln Sea flux gate. **b** The annual mean sea ice thickness (m) over the southern Lincoln Sea estimated from PIOMAS data. **c** The annual mean ice volume flux (km^3^) through the southern Lincoln Sea flux gate. The data in red is from Kwok et al study with the data in blue from this study. Means over the period of the two data sets (1997–2009) and (2016–2019) are shown with dashed lines. Annual means defined from Sept 1 to Aug 31. In **b** and **c**, error bars are included based on the uncertainty in ice thickness derived from the comparison with CryoSat2 data shown in Fig. 7 (see Methods). As discussed in the Methods Section, the uncertainty in ice area flux is negligible on annual time scales.

The annual mean ice area flux time series (Fig. 8a) indicates that the average over the 2017–2019 exceeded the largest flux previously observed, that occurred during 2007 when no arches formed^2^. Furthermore, over the period 1997–2009 the average annual mean ice area flux was 42,000 km^2^ while over 2017–2019 it was over twice as large at 86,000 km^2^. Arctic sea ice is becoming thinner, this is also true for the Lincoln Sea where the PIOMAS annual mean ice thickness has decreased from 3.7 m during the period 1997–2009 to 2.4 m recently with an uncertainty of ±0.75 m (Fig. 8b). The ice volume flux can be estimated from the product of the ice area flux and Lincoln Sea ice thickness. This time series is shown in Fig. 8c. Unlike the situation that occurred for the ice area flux, the ice volume flux during 2007 was higher than that for any of the years from 2017 to 2019. This is the result of the recent thinning of the Lincoln Sea ice cover. However, the average annual ice volume flux over the period 2017–2019 was nevertheless ~70% larger, at 190 ± 55 km^3^, than that for the period 1997–2009, 112 ± 16 km^3^.

In addition to the aforementioned changes with time, all three-time series shown in Fig. 8 indicates the presence of inter-annual variability that may be associated with variability in sea ice motion across the central Arctic that has been shown to impact sea ice thickness in the Lincoln Sea^14^. We note that the difference in the mean ice thickness and ice volume flux between the two periods under investigation exceeds the corresponding uncertainty suggesting that the changes are robust.

### Ice Arch Stability

**Fig. 9:**
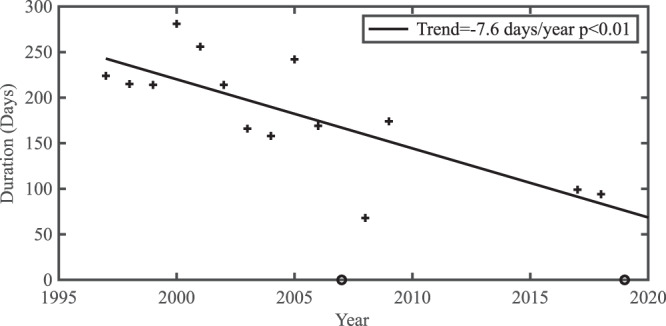
Time series of duration (days) of ice stoppage along Nares Strait. During 2007 and 2019, indicated by the ‘o’, no arches formed. The linear least squares fit to the data is also shown along with the trend and statistical significance.

Finally, we assess the time evolution of the stability of the Nares Strait ice arches by combining their duration as previously reported^2^ with that derived for 2016–2019 from the ice area flux data presented herein. Figure 9 presents the results for the combined duration of both the northern and southern ice arches with a significance test described in the Methods Section. Similar results were obtained when the two arches were considered separately. Over time, the tendency for shorter duration arches is evident with a trend of approximately −7 days/year (*p* < 0.01).

## Discussion

The largest loss of Arctic sea ice occurs through Fram Strait^31^, on the east side of north Greenland, with typical annual ice area fluxes on the order of 900,000 km^2^. Although there has been a similar recent increase in ice area flux through Fram Strait, again indicative of a more mobile ice pack, there has been no corresponding increase in ice volume flux^31^. Given that the ice volume flux is the product of the ice area flux and the ice thickness, this implies that for the Nares Strait region the increase in ice area flux exceeds the reduction in thickness with the two compensating more or less for the Fram Strait region.

Recent work indicates that ice motion in the Last Ice Area, that includes the Lincoln Sea, is increasing at twice the rate as the entire Arctic Ocean^14^. In addition, a number of theoretical^16,32^ and observational^8,17^ studies have proposed that the stability of the Nares Strait ice arches decreases with thinning ice cover. These results are consistent with those presented herein all of which provide additional evidence of the changing nature of the Arctic as we transition to a thinner more mobile ice pack. Results of this study also highlight that with continued Arctic warming, ice arch stability in Nares Strait as well as throughout the adjacent CAA will decrease resulting in more frequent transport of Arctic Ocean multi-year to southerly latitudes^7^, that will have negative implications for the maritime industry^33,34^ as well as impacting food security and other traditional activities for indigenous communities in the Arctic^35^.

The current configuration of the North Water Polynya, as a latent heat polynya, depends on the presence of the Nares Strait ice arches^36^ to restrict the southward flux of thick multi-year ice along Nares Strait. This allows the strong winds and ocean currents that occur in the vicinity of Smith Sound^23,37^ to advect thin ice away allowing the polynya to form. It follows that a weakening of the Nares Strait ice arches may impact the North Water Polynya leading to regional changes in primary and secondary production that will be felt throughout the entire food chain

## Methods

### Ice Area Flux

Annual (September to August) ice area flux through Nares Strait for 2016–2019 was determined using an established technique^2,18,38^. First, sea ice motion from each sequential pair of Sentinel-1 imagery (~0.5 to 1-day time separation) was determined using the Komarov and Barber tracking algorithm^39^. Sea ice motion was then interpolated to a 30 km buffer region surrounding the gate and sampled at 5 km intervals across. Considering that ice rapidly deforms as it is being funneled through Nares Strait we placed our gate farther north of Nares Strait than has been done previously^2^ to facilitate improved motion detection. The ice area flux (F) was calculated using: $F = {\sum} {c_iu_i\Delta x}$ where c_i_ is the ice concentration obtained from the closest Canadian Ice Service ice chart^40^ to the Sentinel-1 image date, u_i_ is the ice speed normal to the flux gate at the ith location and Δx is the spacing along the gate (5 km). If we assume that the errors of the sea ice motion samples are additive, unbiased, uncorrelated, and normally distributed, then the uncertainty in ice area flux across the gate (σ_f_) can be determined using the following equation: $\sigma _f = \sigma _eL\left( {\sqrt {N_s} } \right)^{ - 1},$where, σ_e_ is the error in ice motion of 0.43 km/day determined previously^39^, L is the width of the gate and Ns is the number of samples across the gate. For L=139 km and Ns=27 the uncertainty in ice area flux at our gate is ~±12 km^2^/day. On monthly or annual timescales, the uncertainty is close to zero.

### Statistical Significance

Many geophysical time series are characterized by red-noise arising from temporal autocorrelation that results in power spectra that have elevated power at low frequencies^41,42^. To account for this characteristic, which if unaccounted for may result in an overestimation of the significance^42^, we use is a non-parametric Monte Carlo technique where a large number, in this instance 100,000, of synthetic time series that share the same power spectrum as the original time series are generated. The distribution of the trends of these synthetic time series are used to estimate the significance of the trend in the underlying time series. By sharing the same spectral characteristics, one has greater confidence that they share the same background variability and one is not introducing a bias into the assessment of the statistical significance of the trend. To generate the synthetic time series, the Fast Fourier Transform (FFT) of the underlying time series is calculated. For each individual synthetic time series, the phase of the Fourier components are randomized and then the Inverse FFT is calculated^41^.

## Data Availability

Sentinel-1 SAR imagery is available from the Copernicus Open Access Hub at: http://scihub.copernicus.eu. AMSRE/2 sea ice concentration data is available from the University of Bremen at: https://seaice.uni-bremen.de/. The PIOMAS sea ice thickness data is available from the Polar Sciences Center at the University of Washington at: http://psc.apl.uw.edu/research/projects/arctic-sea-ice-volume-anomaly/. The AWI CryoSat2 sea ice thickness data is available from the Alfred Wegener Institute at: http://data.meereisportal.de/data/cryosat2/version2.2/. The UCL CryoSat2 sea ice thickness data is available from the University College London at: http://www.cpom.ucl.ac.uk/csopr/seaice.html. Moore^43^ provides the ice area flux time series derived from the Seninel-1 data.

## References

[1] Kwok, R. Variability of Nares Strait ice flux. *Geophys. Res. Lett.* **32** (2005).

[2] Kwok R, Toudal Pedersen L, Gudmandsen P, Pang SS (2010). Large sea ice outflow into the Nares Strait in 2007. Geophys. Res. Lett..

[3] Kozo TL (1991). The hybrid polynya at the northern end of Nares Strait. Geophys. Res. Lett..

[4] Bourke RH, Garrett RP (1987). Sea ice thickness distribution in the Arctic Ocean. Cold Reg. Sci. Technol..

[5] Kwok R (2018). Arctic sea ice thickness, volume, and multiyear ice coverage: losses and coupled variability (1958–2018). Environ. Res. Lett..

[6] Maslanik, J., Stroeve, J., Fowler, C. & Emery, W. Distribution and trends in Arctic sea ice age through spring 2011. *Geophys. Res. Lett.* **38**, 10.1029/2011GL047735 (2011).

[7] Melling, H. Sea ice of the northern Canadian Arctic Archipelago. *J. Geophys. Res.: Oceans* **107**, 2-1-2-21 (2002).

[8] Kwok, R. et al. Thinning and volume loss of the Arctic Ocean sea ice cover: 2003–2008. *J. Geophys. Res.: Oceans* **114** (2009).

[9] Ryan PA, Münchow A (2017). Sea ice draft observations in Nares Strait from 2003 to 2012. J. Geophys. Res.: Oceans.

[10] Barber DG, Hanesiak JM, Chan W, Piwowar J (2001). Sea‐ice and meteorological conditions in Northern Baffin Bay and the North Water polynya between 1979 and 1996. Atmosphere-Ocean.

[11] Münchow A (2016). Volume and Freshwater Flux Observations from Nares Strait to the West of Greenland at Daily Time Scales from 2003 to 2009. J. Phys. Oceanogr..

[12] Laliberté F, Howell SEL, Kushner PJ (2016). Regional variability of a projected sea ice-free Arctic during the summer months. Geophys. Res. Lett..

[13] Sou T, Flato G (2009). Sea Ice in the Canadian Arctic Archipelago: modeling the past (1950–2004) and the future (2041–60). J. Clim..

[14] Moore, G.W.K., Schweiger, A., Zhang, J. & Steele, M. Spatiotemporal variability of Sea Ice in the Arctic’s Last Ice Area. *Geophys. Res. Lett.* **46**, 11237–11243 (2019).

[15] Pfirman S, Tremblay B, Newton R, Fowler C (2010). The last Arctic sea ice refuge. AGUFM.

[16] Hibler W, Hutchings J, Ip C (2006). Sea-ice arching and multiple flow states of Arctic pack ice. Ann. Glaciol..

[17] Moore GWK, McNeil K (2018). The Early Collapse of the 2017 Lincoln Sea Ice Arch in Response to Anomalous Sea Ice and Wind Forcing. Geophys. Res. Lett..

[18] Howell SE (2013). Recent changes in the exchange of sea ice between the Arctic Ocean and the Canadian Arctic Archipelago. J. Geophys. Res.: Oceans.

[19] Samelson, R.M., Agnew, T., Melling, H. & Münchow, A. Evidence for atmospheric control of sea‐ice motion through Nares Strait. *Geophys. Res. Letters* **33** (2006).

[20] Hansen, K. *Ice Arch Crumbles Early*, https://earthobservatory.nasa.gov/images/145232/ice-arch-crumbles-early (2019).

[21] Spreen G, Kaleschke L, Heygster G (2008). Sea ice remote sensing using AMSR-E 89-GHz channels. J. Geophys. Res.: Oceans.

[22] Dumont D, Gratton Y, Arbetter TE (2010). Modeling Wind-Driven Circulation and Landfast Ice-Edge Processes during Polynya Events in Northern Baffin Bay. J. Phys. Oceanogr..

[23] Moore GWK, Våge K (2018). Impact of model resolution on the representation of the air–sea interaction associated with the North Water Polynya. Q. J. R. Meteorological Soc..

[24] Münchow A, Melling H (2008). Ocean current observations from Nares Strait to the west of Greenland: Interannual to tidal variability and forcing. J. Mar. Res..

[25] Schweiger A (2011). Uncertainty in modeled Arctic sea ice volume. J. Geophys. Res.: Oceans.

[26] Zhang J, Rothrock DA (2003). Modeling Global Sea Ice with a Thickness and Enthalpy Distribution Model in Generalized Curvilinear Coordinates. Monthly Weather Rev..

[27] Howell SE, Laliberté F, Kwok R, Derksen C, King J (2016). Landfast ice thickness in the Canadian Arctic Archipelago from observations and models. Cryosphere.

[28] Lindsay R, Schweiger A (2015). Arctic sea ice thickness loss determined using subsurface, aircraft, and satellite observations. Cryosphere.

[29] Ricker R, Hendricks S, Helm V, Skourup H, Davidson M (2014). Sensitivity of CryoSat-2 Arctic sea-ice freeboard and thickness on radar-waveform interpretation. Cryosphere.

[30] Tilling RL, Ridout A, Shepherd A (2018). Estimating Arctic sea ice thickness and volume using CryoSat-2 radar altimeter data. Adv. Space Res..

[31] Smedsrud LH, Halvorsen MH, Stroeve JC, Zhang R, Kloster K (2017). Fram Strait sea ice export variability and September Arctic sea ice extent over the last 80 years. Cryosphere.

[32] Rallabandi B, Zheng Z, Winton M, Stone HA (2017). Formation of sea ice bridges in narrow straits in response to wind and water stresses. J. Geophys. Res.: Oceans.

[33] Barber, D. et al. Increasing mobility of high Arctic sea ice increases marine hazards off the east coast of Newfoundland. *Geophys. Research Letters* (2018).

[34] Haas C, Howell SE (2015). Ice thickness in the Northwest Passage. Geophys. Res. Lett..

[35] Huet C (2017). Food insecurity and food consumption by season in households with children in an Arctic city: a cross-sectional study. BMC Public Health.

[36] Ingram RG, Bâcle J, Barber DG, Gratton Y, Melling H (2002). An overview of physical processes in the North Water. Deep Sea Res. Part II: Topical Stud. Oceanogr..

[37] Shroyer EL, Samelson RM, Padman L, Münchow A (2015). Modeled ocean circulation in Nares S trait and its dependence on landfast‐ice cover. J. Geophys. Res.: Oceans.

[38] Kwok, R. Exchange of sea ice between the Arctic Ocean and the Canadian Arctic Archipelago. *Geophys. Res. Lett.* **33** (2006).

[39] Komarov AS, Barber DG (2013). Sea ice motion tracking from sequential dual-polarization RADARSAT-2 images. IEEE Trans. Geosci. Remote Sens..

[40] Tivy, A. et al. Trends and variability in summer sea ice cover in the Canadian Arctic based on the Canadian Ice Service Digital Archive, 1960–2008 and 1968–2008. *J. Geophys. Res.: Oceans* **116** (2011).

[41] Rudnick DL, Davis RE (2003). Red noise and regime shifts. Deep Sea Res. Part I: Oceanographic Res. Pap..

[42] Santer BD (2000). Statistical significance of trends and trend differences in layer-average atmospheric temperature time series. J. Geophys. Res.: Atmospheres.

[43] Moore, G.W.K. Nares Strait Ice Area Flux, 10.5683/SP2/WRGX0K, (2019).

